# Evaluating the Efficacy of Volume Isotropic Turbo Spin Echo Acquisition Versus T2-Weighted Turbo Spin Echo Imaging in the Diagnosis of Nerve Root and Perineuronal Pathologies in Spinal Disorders

**DOI:** 10.7759/cureus.60988

**Published:** 2024-05-24

**Authors:** Ajina Sam, Jaypradha S, Vinoth Pandian, Karthik Krishna Ramakrishnan, Paarthipan Natarajan, Yuvaraj Muralidharan

**Affiliations:** 1 Department of Radiology, Saveetha Medical College and Hospital, Saveetha Institute of Medical and Technical Sciences (SIMATS), Saveetha University, Chennai, IND; 2 Department of Radiodiagnosis, Saveetha Medical College and Hospital, Chennai, IND

**Keywords:** nerve root and perineuronal pathology, mri, vista, isotropic turbo spin echo, 3d isotropic tse

## Abstract

Background

While two-dimensional (2D) turbo spin echo (TSE) sequences offer better through-plane resolution than three-dimensional (3D) isotropic TSE sequences images, with a narrower thickness of the slice, 3D isotropic TSE sequences are known to have a weaker in-plane resolution as well as blurring of the image. These elements may make it more difficult to distinguish between nearby structures that may affect nerve roots and small nerve roots during spinal imaging. This study aimed to analyze the accuracy of T2 TSE sequence and volumetric isotropic TSE acquisition in determining the indentation of nerve roots and perineural diseases such as nerve sheath tumors and Tarlov cysts.

Methods

Fifty patients who attended the Department of Radiodiagnosis for magnetic resonance (MR) spine participated in this prospective study. Routine MR lumbosacral (LS) spine sequences, such as survey, coronal T2 short-tau inversion recovery (STIR), sagittal T2 TSE, sagittal T1 TSE, and axial T2 TSE, were carried out after a localizer was acquired. More sequences from volume isotropic turbo spin echo acquisition (VISTA) were acquired. For both 2D and 3D sequences, the visibility ratings for perineural cysts, spinal canal stenosis, and nerve root indentation were evaluated. Visibility ratings ranged from zero to four.

Results

In the cases of perineural cyst, spinal canal stenosis, and nerve root impingement, the mean difference between the VISTA and T2 TSE visibility scores was 0.04, 0.54, and 0.56, respectively. The VISTA and T2 TS had standard deviation differences of 0.006, 0.026, and 0.06, respectively. The "t" values for nerve root impingement, spinal canal stenosis, and perineural cysts were, in order, 50, 180, and 70. Because the p-value was <0.01, a statistically significant variation has been observed.

Conclusion

In the diagnosis of neural and perineuronal disorders, the visibility scores for 3D T2 TSE (VISTA) were considerably better than those for 2D T2 TSE in identifying perineural cysts, spinal canal stenosis, and nerve root indentation.

## Introduction

Two-dimensional (2D) T2-weighted (T2W) turbo spin echo (TSE) sequences are used in the current lumbar spine (LS) magnetic resonance imaging (MRI) technique, yet they have a number of drawbacks. First, oblique planes with a certain orientation are required in complex anatomy such as lordosis and scoliosis disorders in order to see a specific feature that would otherwise be difficult to see. In this instance, imaging various planes requires more time. Second, some minor structures, which are nerve root compressions, which could be the primary cause of lower back pain or even certain lesions, might be missed in these images due to the restricted ability to choose slice thickness in the 2D TSE images as well as larger interslice gaps.

Decreased partial volume artifacts also had an impact on the 2D TSE nerve root assessment in the extraforaminal zone. At the extraforaminal zone, the nerve that leaves the neural foramen dips obliquely outward and downward. Due to a partial volume artifact, the root of the nerve immediately above or below the ruptured extraforaminal disc on a 2D sequence seems to be in the same plane as the disc because of the nerve's oblique course [1,2]. As a result, it is possible to miss the real touch or to overlook the fact that has been retained between the disc and nerve root. Employing the three-dimensional (3D) isotropic T2W TSE sequence to capture coronal along with the oblique coronal images with an expanded field of view facilitated the identification of the extraforaminal nerve root as well as the adjacent tissues in the longitudinal plane. The limited extent of the sagittal imaging plane in the conventional 2D TSE sequence limits the extraforaminal zone evaluation. While 2D TSE sequences offer better through-plane resolution than 3D isotropic TSE sequence images with a narrower thickness of the slice, 3D isotropic TSE sequences are known to have weaker in-plane resolution along with the image blurring [3-6]. These elements may make it more difficult to distinguish between nearby structures that may affect nerve roots and little nerve roots during spinal imaging. This study aimed to analyze the accuracy of T2 TSE sequence and volumetric isotropic TSE acquisition in determining the indentation of nerve roots and perineural diseases such as nerve sheath tumors and Tarlov cysts.

## Materials and methods

Study design

This prospective study was conducted over a six-month period at Saveetha Medical College and Hospital, aiming to evaluate spine pathologies through MRI. A total of 50 patients were included, with the sample size determined based on the expected prevalence of spine conditions requiring MRI during the study period to ensure robust statistical analysis.

Sample Size and Calculation

The study included 50 patients, with the sample size calculated based on the anticipated prevalence of spine pathologies needing MRI within the study timeframe. This calculation was designed to facilitate a robust statistical analysis of the results.

Inclusion Criteria

**Figure 1:**
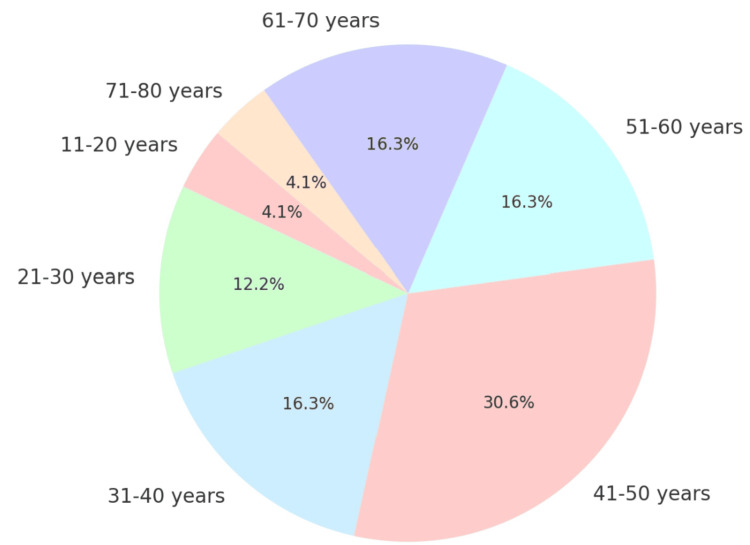
Pie chart depicting patient distribution by age

The study included patients referred to the Department of Radiodiagnosis for MRI spine, encompassing a range of age groups as shown in Figure 1 with various spine conditions. Specifically, patients presenting with nerve root indentations, nerve impingements, nerve sheath tumors, and cysts were included.

Exclusion Criteria

The exclusion criteria for this study were specifically designed to mitigate any risks that could compromise the safety and integrity of the MRI. Patients with a history of claustrophobia, those with metallic implants, cardiac pacemakers, or metallic foreign bodies in situ, and clinically unstable patients were excluded from the study to ensure the safety and reliability of the imaging process.

Ethical Approval

Ethical approval for the study was obtained from the Institutional Review Board (IRB) of Saveetha Medical College and Hospital, with the approval number SCAHS/IRB/2022/March 215. Written informed consent was obtained from each participant prior to their inclusion in the study.

Patient Preparation

Patients were prepared for the MRI by removing all ferromagnetic materials and recording their weight. They were provided with detailed instructions regarding the MRI procedure, and the verification of each patient’s unique hospital ID was conducted to ensure accurate identification.

MRI Protocol

The MRI was performed using a Philips Multiva 1.5T scanner with a magnetic field strength of 1.5 Tesla and a total image matrix (TIM) coil. The routine imaging sequences included survey, coronal T2 short-tau inversion recovery (STIR), sagittal T2 TSE, sagittal T1 TSE, and axial T2 TSE. Additionally, the volume isotropic turbo spin echo acquisition (VISTA) sequence was utilized for enhanced imaging.

Imaging Parameters, Process, and Analysis

For the VISTA sequence, the parameters were set with a repetition time (TR) of 2000 ms, an echo time (TE) of 200 ms, an acquisition time (TA) of five minutes, and a flip angle (FA) of 90°. The reconstruction matrix was 256, with a 3D scan mode and a TSE factor of 94. Patients were positioned supine with the landmark at the symphysis menti. A three-plane localizer, typically lasting less than 25 seconds, was initially taken to plan the sequences. Sequence planning for the coronal STIR included slices from the T11 to the coccyx, encompassing the lateral borders of the right and left transverse processes. Similarly, sagittal T2W slices covered from T11 to the coccyx, including the lateral borders of the right and left transverse processes. Image analysis was performed using the Medsynapse assessment tool, focusing on parameters such as the visibility of perineural cysts, spinal canal stenosis, and nerve root indentations. Visibility scores ranged from 0 to 4, with 0 indicating nonvisibility; 1, barely visible; 2, adequately visible; 3, good visibility; and 4, excellent visibility.

Statistical analysis

The statistical analysis of the study was meticulously planned to assess the effectiveness of the 3D VISTA sequence compared to the traditional 2D T2 TSE sequence. Key components of the analysis include visibility score analysis, descriptive statistics like mean and standard deviation, and inferential statistics like paired t-tests and statistical significance (p-value).

## Results

Thirty of the 50 cases were men, and 20 were women. Two males were in the 11-20 age group; three females and three males were in the age group of 21-30; three females and five males were in the age group of 31-40; six females and nine males were in the age group of 41-50; five females and three males were in the 51-60 age group; three females and five males were in the age group of 61-70; and two males were in the age group of 71-80 as shown in Table 1.

**Table 1:** Patient distribution based on age group

Age group	Number of patients		Total
	Male	Female	
11-20 years	2	-	2
21-30 years	3	3	6
31-40 years	5	3	8
41-50 years	9	6	15
51-60 years	3	5	8
61-70 years	5	3	8
71-80 years	2	-	2

**Figure 2:**
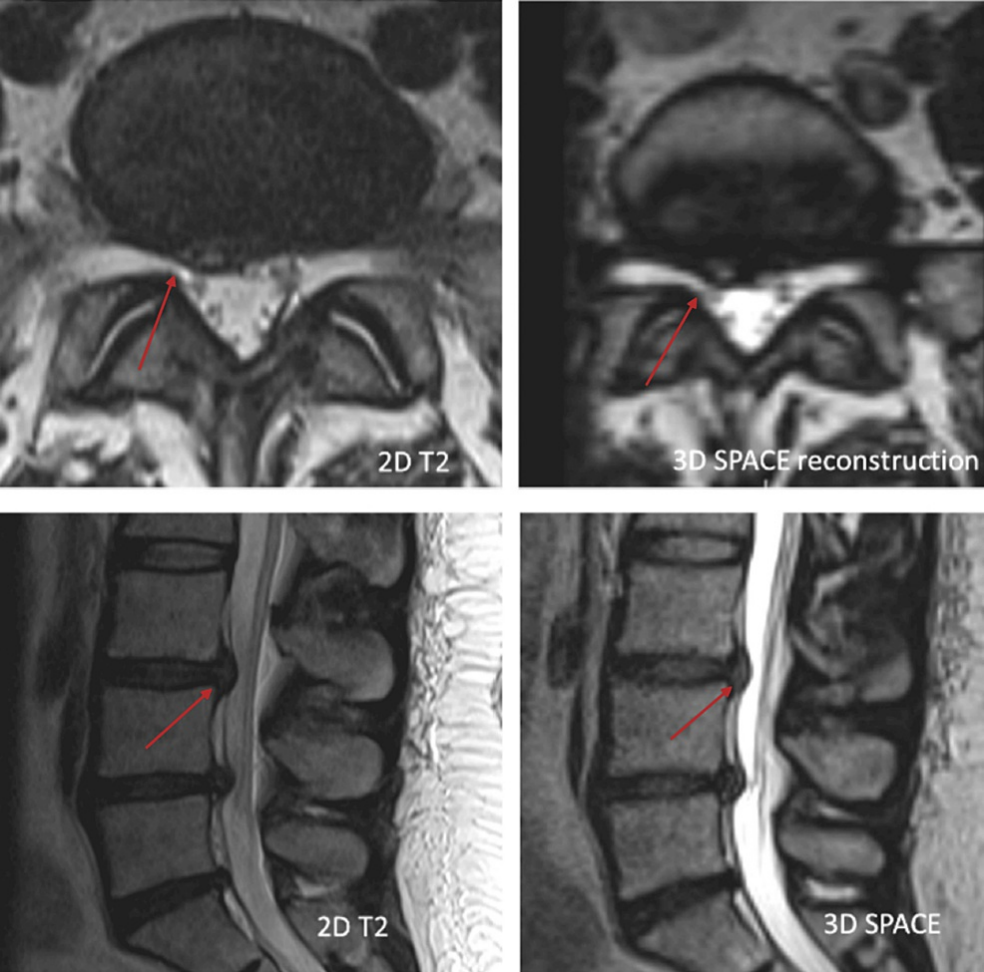
Comparison of 2D MRI and 3D SPACE axial (top row) and sagittal lumbar spine MRI of the same patient. Although higher in-plane resolution of 2D sequences gives crisper images, the presence and severity of spinal canal stenosis can be determined as obviously on SPACE as on the 2D sequences 2D: Two-dimensional; MRI: magnetic resonance imaging; 3D: three-dimensional; SPACE: Sampling Perfection with Application-optimized Contrasts using different flip angle Evolution

**Figure 3:**
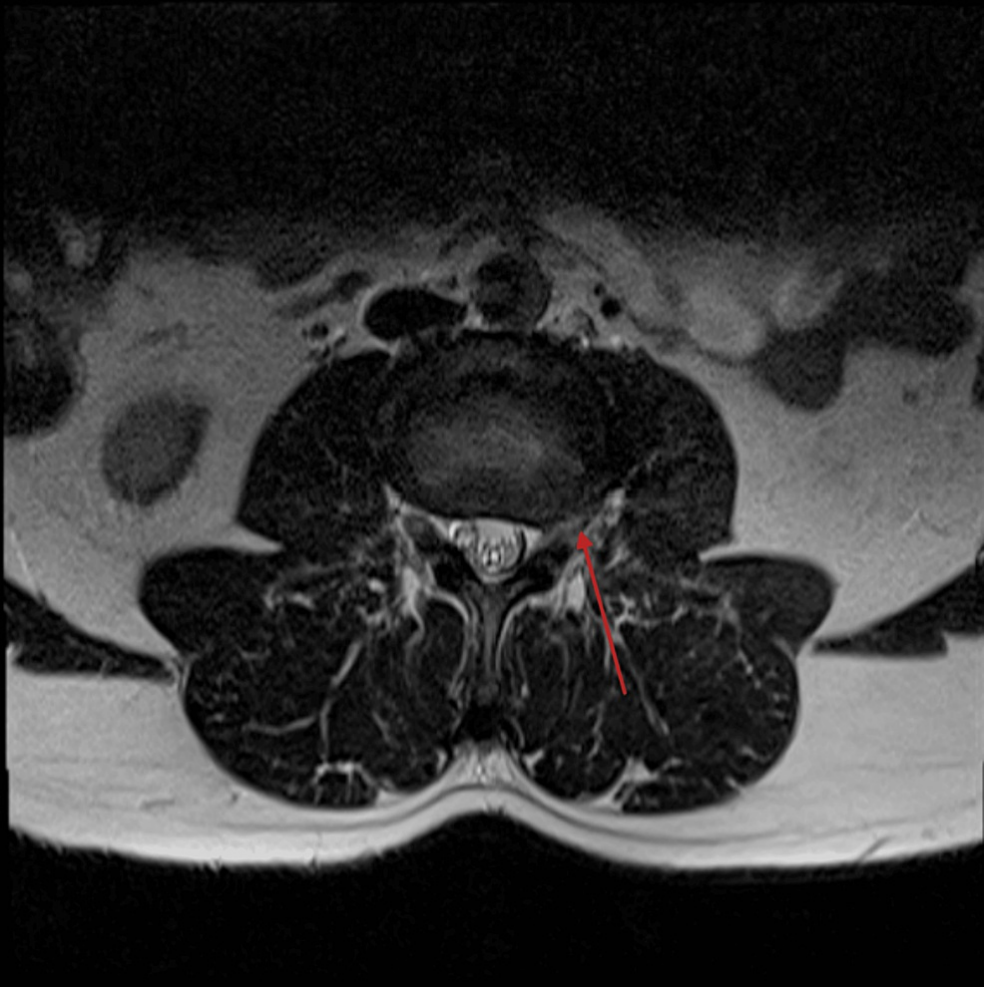
MRI spine axial section T2 sequence showing small (<1 cm) left far foraminal disc at L3/L4, which is impinging on the exiting L3 nerve just after it enters the exit foramen.  Image source: Case courtesy of Ian Bickle, Radiopaedia.org. From the case https://radiopaedia.org/cases/44881?lang=gb rID: 44881 MRI: Magnetic resonance imaging

**Figure 4:**
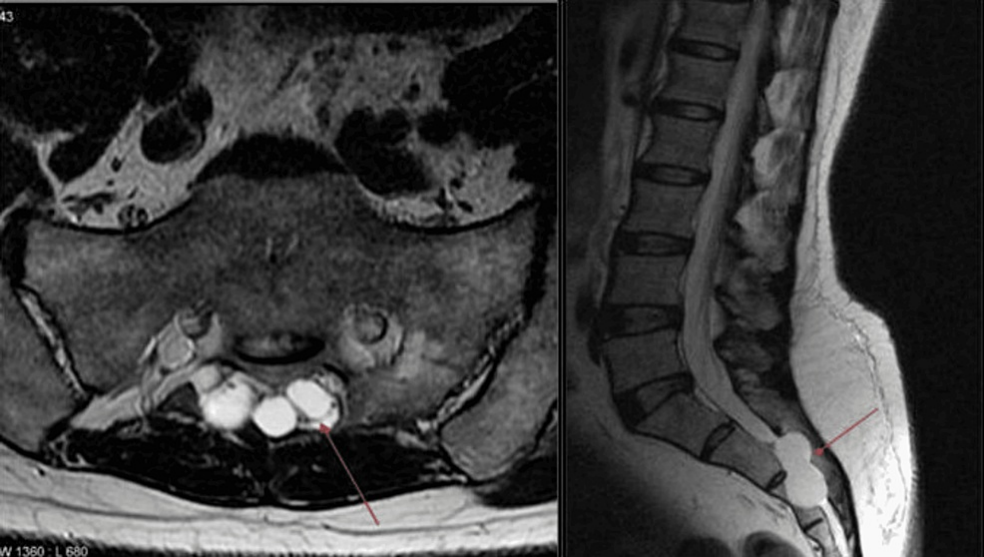
Axial and sagittal MRI T2 images of the spine showing perineural cysts. Image source: Case courtesy of Frank Gaillard, Radiopaedia.org. From the case https://radiopaedia.org/cases/6284?lang=gb rID: 6284

Of the 50 instances, 29 had spinal canal stenosis as shown in Figure 2, 23 had nerve root indentation as shown in Figure 3, and one patient had a perineural cyst as shown in Figure 4. Of the 23, six had nerve root indentation in the ventral sac, four had it crossing the nerve roots, eight had it in a neural foramen, and five had it leaving the nerve roots as shown in Tables 2-3.

**Table 2:** Patient distribution based on pathologies

Pathology	No of cases
Nerve root indentation	23
Spinal canal stenosis	29
Perineural cyst	1

**Table 3:** Patient distribution based on the classification of nerve root indentation

Classification	No of cases
Ventral sac	6
Traversing nerve roots	4
Neural foramen	8
Exiting nerve roots	5

In the VISTA and T2 TSE sequences, the mean visibility scores for perineural cysts were 0.08 and 0.04, respectively, as shown in Figure 6. In the case of spinal canal stenosis, the VISTA and T2 TSE sequences showed values of 2.32 and 1.78, respectively. In the VISTA and T2 TSE sequences, it was 1.84 and 1.28 in nerve root impingement, respectively. In the VISTA and T2 TSE sequences, the standard deviation for the perineural cyst was 0.01 and 0.005, respectively. In the VISTA and T2 TSE sequences, they were, respectively, 0.13 and 0.16 in cases with spinal canal stenosis. It was 0.08 in the T2 TSE sequence and 0.14 in the VISTA sequence for nerve root impingement. In the cases of perineural cyst, spinal canal stenosis, and nerve root impingement, the mean difference between the VISTA and T2 TSE was 0.04, 0.54, and 0.56, respectively. In the cases of perineural cyst, spinal canal stenosis, and nerve root impingement, the standard deviation difference between the VISTA and T2 TS was 0.006, 0.026, and 0.06, respectively.

**Figure 5:**
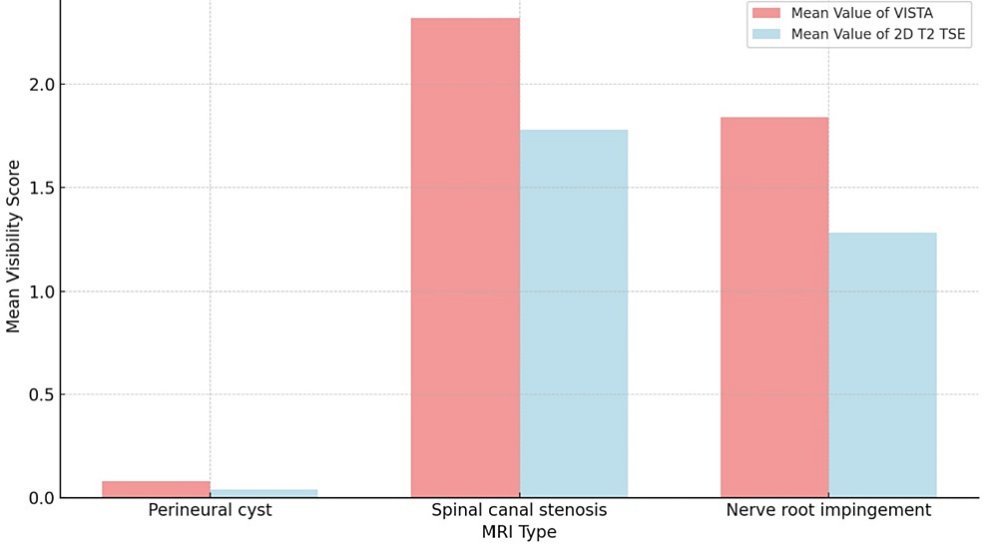
Bar graph depicting the comparison of mean visibility score by MRI type MRI: Magnetic resonance imaging

In this study, the difference in diagnostic value between T2 TSE and VISTA was analyzed by utilizing a paired t-test. The "t" values for perineural cyst, spinal canal stenosis, and nerve root impingement were, in accordance with the paired T-test, 50, 180, and 70, respectively. After obtaining the "t" value, the p-value was determined. When the result is statistically significant at p < 0.05, the p-value is less than 0.01 as shown in Table 4 and Figure 5.

**Table 4:** Represents the mean value of visibility score with respect to sequences and pathologies MRI: Magnetic resonance imaging; SD: standard deviation; 2D: two dimensional; TSE: turbo spin echo; VISTA: volume isotropic turbo spin echo acquisition The p-value (α = 0.05) in this table underscores the superiority of VISTA over 2D T2TSE for each pathology. A p-value less than 0.05 signifies a statistically significant difference at the 5% significance level, demonstrating VISTA's efficacy over 2D T2TSE for spinal pathologies

Pathologies	Mean value of VISTA	Mean value of 2D T2TSE	SD of VISTA	SD of 2D T2 TSE	Mean difference	SD difference	T value	p-value (α = 0.05)
Perineural cyst	0.08	0.04	0.01	0.005	0.04	0.006	50	<0.01
Spinal canal stenosis	2.32	1.78	0.13	0.16	0.54	0.026	180	<0.01
Nerve root impingement	1.84	1.28	0.14	0.08	0.56	0.06	70	<0.01

## Discussion

Several axial imaging techniques, including the stack and through-disc approaches, are needed for the traditional 2D TSE MRI acquisition procedure. Individual acquisition of independent examination planes is also required. This results in longer acquisition durations, the requirement for manual imaging orientation, less-than-ideal planes, and the potential for pathology to be missed in the interim between acquisitions [7]. Three-dimensional TSE MRI can offer multi-planar reconstruction in comparison to traditional 2D TSE MRI, which shortens the scanning time for the patient [7]. When compared to traditional 2D T2W sequences, previous phantom studies of healthy subjects undergoing 3D-TSE MRI examination showed an enhanced SNR and visualization of the nerve roots, intra-foraminal structures, and neural foramina [8,9]. For high-resolution, contiguous, thin-section isotropic images of the intricate anatomy of this body part, 3D isotropic techniques like CUBE, VISTA, and sampling perfection with the application-optimized contrasts by utilizing various FA evolutions (Sampling Perfection with Application optimized Contrast using different flip angle Evolution (SPACE)) were utilized in a clinical application variety. The benefits of 3D isotropic T2W imaging (T2WI) sequences in the lumbar spine have been documented in numerous investigations [10].

Comparing the axial 3D TSE sequence to the traditional 2D fast spin echo (FSE), there are improvements in contrast-to-noise ratio (CNR) and signal-to-noise ratio (SNR), picture contrast, flow artifact reduction, and visibility of fine anatomical structures [11]. The accuracy of diagnosing disc herniation, spinal canal stenosis, and degenerative changes in the cervical spine was similar in another investigation that used 3D and 2D TSE T2WI. One benefit of the 3D TSE T2WI is that reprocessing is easier, especially when determining whether pathology or deformity is present. A relatively new MRI approach that is accessible on some MR machines is the 3D TSE approach. This sequence can be used in place of or in addition to standard 2D-FSE sequences for lumbar imaging (as a problem solver) [4,6]. It also has certain other features, such as the ability to dynamically modify the imaging plane by utilizing an add-on program to enhance the functionality of the default image-viewing software [6]. Several researchers have examined the technical picture quality of 2D as well as 3D MRI sequences for the cervical spine in the literature [8,9]. To find out if the 3D SPACE sequence is appropriate for routine spine imaging, Tins et al. attempted to apply the SPACE sequence in the 62 MRI exams with two examiners [12]. In their investigation, 84% o=the instances had extremely acceptable anatomical depictions, with a high interobserver agreement for the SPACE sequence.

For every pathology found in the current investigation, the visibility scores in the VISTA sequence were greater than those in the T2 TSE sequence. There was a statistically significant difference. Comparing higher-resolution 3D T2W TSE imaging to standard-resolution 2D T2W TSE imaging, Sartoretti et al. demonstrated that more nerve roots had high-grade nerve root compression and foraminal stenoses in the lateral recesses [13]. The potential utility of higher-resolution 3D imaging in the identification of spinal disease along with nerve root impairment has been evaluated in earlier research [2,14-17]. For instance, in order to diagnose lumbar nerve root impairment, Sung et al. compared a typical 2D T2W sequence having a higher-resolution, isotropic 3D T2W TSE SPACE sequence [11]. It is interesting to note that when comparing their high-resolution 3D sequencing to their regular 2D sequence, the investigators found no discernible benefit in diagnosing nerve root impairment [17].

Eighty patients underwent the 3D TSE sequence, and the outcomes have been compared with the 2D-FSE sequence by Blizzard et al. [9]. By utilizing 57 criteria, such as central canal stenosis along with disc herniation, point-by-point agreement was used to calculate inter method reliability for each interpreter. Overall inter-method reliability in their study was 85.3%, and modified reliability, which does not include discrepancies between moderate abnormalities and normal, was 94.6%. A 3.0T MR scanner was used by Lee et al. to investigate lumbar spinal MRI [2]. They used two interpreters to compare 2D T2W TSE and 3D T2W SPACE sequences for lumbar neural foraminal stenosis, nerve compression, and central spinal stenosis. There has been no variation among the 2D T2W SPACE and 3D T2W TSE sequences in how well they found foraminal stenosis at 32 foramen levels (78.9% versus 78.9%). Both the sequences showed 100% sensitivity at the 42 spinal levels for spinal stenosis, and 92.9% and 81.8% sensitivity ratios for 3D T2W TSE and 2D T2W, respectively, at 59 spinal nerves for nerve compression.

In a study on T2W 3D isotropic TSE (3D-SPACE) versus 2D T2W TSE sequences for the lumbar spine MRI, Hossein et al. observed that the 3D SPACE T2W sequence significantly improved visibility, CNR, and SNR (p-value < 0.05) [16]. This observation aligned with the current investigation. The study also found that the interobserver diagnostic value agreement for the visibility of regions of interest has been substantial and flawless (k > 0.6). Also, for every pathologic index (k >0.6), there was a significant and flawless agreement between the observers and methods. Compared to the 2D TSE sequence (k = 0.603), the 3D SPACE sequence had a greater interobserver agreement (k = 0.793). In the axial, sagittal, and coronal planes, the 3D SPACE sequence's multi-planar reconstructions (MPR) time of scan was less than that of the 2D TSE (209 s) at 192 s. Lower NU values of the muscles and cerebrospinal fluid (CSF) were found in 2D T2W sagittal sequences than in the 3D VISTA sequence, according to a quantitative study by Kwon et al. [8]. Between the 2D TSE and 3D VISTA sequences, there has been no statistically significant difference, as per the other NU values (0.059 < p < 0.959). Comparing 3D VISTA pictures to 2D sequences, the former had significantly fewer CSF flow artifacts (p < 0.001) and more clearly defined neural foramina (p = 0.016) and intradural nerve rootlets (p = 0.001). They concluded that for the purpose of demarcating intradural nerve rootlets along with neural foramina, the 3D T2W sequence is more effective than traditional 2D sequences.

According to a study on 3D T2-SPACE diagnostic quality, Chokshi et al. found that for both reviewers, the average visibility scores for the intervertebral disc signal, ligamentum flavum, neural foramina, dorsal rootlets, and ventral rootlets have been greater for T2-SPACE than with the T2-FSE in the cervical spine MRI anatomy assessment (P < .001) [17]. The average ratings for the other structures either did not depict a statistically significant variation or the reviewers' opinions about which sequence was superior varied. Less CSF flow artifact was seen in T2-SPACE (p < .001). The interobserver variability for T2-FSE was −0.02-0.30, while for T2-SPACE, it was −0.02-0.20. Jinkyeong Sung [14] discovered that using a 3D isotropic T2W TSE sequence to evaluate nerve root compromise in the lumbar spine did not show significant differences compared to using 2D TSE sequences. When 2D and 3D are combined, the diagnostic accuracy may be higher than when they are used alone.

This prospective study aimed to assess the validity of VISTA as well as T2 TSE in the spine in cases of neural and perineuronal disorders. Information loss may result from the larger slice thickness and interslice gap used in the traditional 2D nonisotropic sequence. The outcomes depicted that there were no appreciable variations among the 2D T2 TSE and the 3D T2 TSE (VISTA) in terms of identifying perineural cysts, spinal canal stenosis, and nerve root indentation. Furthermore, it is helpful in determining the cause of the perineural cyst, the depression of the nerve root caused by the cyst, the identification of nerve compression using restructured 3D pictures, and the precise illustration of the nerve root and herniated disc. By using thin, continuous slices to reduce the risks associated with 2D sequences, the 3D isotropic VISTA sequence can be rebuilt into multiple planes, obviating the necessity for multiple-plane acquisition. Better diagnoses are made possible by the thin, continuous slices, which offer comprehensive information regarding Tarlov cysts, nerve sheath tumors, and other conditions. The acquisition time is the study's constraint. A single 3D sequence's acquisition duration is over three times longer than that of a sagittal, coronal, or axial 2D scan. Patient movements may be possible due to extra time or a lengthy scan. This may also result in artifacts and, ultimately, less-than-ideal photos.

Limitations

The sample size of 50 patients may not be sufficient to generalize the findings across a broader population with diverse spinal pathologies. Future studies with larger sample sizes are recommended to validate these results more comprehensively. The assessments of image quality and pathology visibility were based on visual inspection, which is inherently subjective. Despite using a standardized rating scale, interobserver variability might have influenced the results. Employing quantitative imaging metrics could reduce variability and provide more objective assessments in future research. The study did not correlate the imaging findings with clinical outcomes. Understanding whether improvements in imaging translate into better patient outcomes is crucial and should be addressed in subsequent studies. All images were acquired on the same MRI machine using the same settings, which might limit the applicability of the findings to other imaging setups. Future research should consider multicenter studies involving different types of MRI machines to enhance the generalizability of the results. The focus was primarily on specific spinal conditions, potentially overlooking the performance of the imaging techniques with other spinal disorders. Expanding the scope to include a wider range of pathologies could provide a more detailed evaluation of the diagnostic capabilities of the imaging sequences used.

## Conclusions

The 3D T2W VISTA sequence demonstrates clear advantages over the traditional 2D T2W TSE sequence for spinal imaging, particularly in detecting neural and perineuronal pathologies such as spinal canal stenosis, nerve root indentation, and perineural cysts. With statistically significant better visibility scores, the 3D VISTA technique offers more precise and comprehensive visualization of complex spinal structures. This improvement is crucial for accurate diagnosis and effective management of spinal disorders.

Despite the longer acquisition times associated with the 3D technique, which can cause patient discomfort and motion artifacts, the high-resolution capabilities and the ability to obtain multi-planar reconstructions from a single acquisition make it a valuable tool in modern radiology. The study supports integrating 3D VISTA into routine clinical practice to enhance diagnostic accuracy in spinal assessments, pushing forward the boundaries of spinal diagnostics with advanced imaging technology.
